# Long noncoding RNA MyD88 functions as a promising diagnostic biomarker in hepatocellular carcinoma

**DOI:** 10.3389/fendo.2023.938102

**Published:** 2023-01-30

**Authors:** Zhihuai Wang, Peng Gao, Weijun Sun, Adeel ur Rehman, Jiakai Jiang, Suobao Xu, Cailin Xue, Chunfu Zhu, Xihu Qin

**Affiliations:** ^1^ Department of General Surgery, The Affiliated Changzhou No.2 People’s Hospital of Nanjing Medical University, Changzhou, China; ^2^ Graduate School of Nanjing Medical University, Nanjing Medical University, Nanjing, China; ^3^ Chinese Academy of Sciences (CAS) Key Laboratory of Quantitative Engineering Biology, Shenzhen institute of Synthetic Biology, Shenzhen Institute of Advanced Technology, Chinese Academy of Sciences, Shenzhen, China; ^4^ Department of General Surgery, The Changzhou No.3 People’s Hospital, Changzhou, China

**Keywords:** long noncoding RNAs, AFP, diagnostic biomarker, hepatocellular carcinoma, immune infiltration

## Abstract

**Background:**

Hepatocellular carcinoma (HCC) is one of the most frequent malignancies. Alpha-fetoprotein (AFP) has some limitations in diagnosing early HCC. Recently, long noncoding RNAs (lncRNAs) showed great potential as tumor diagnostic biomarkers, and lnc-MyD88 was previously identified as a carcinogen in HCC. Here, we explored its diagnostic value as a plasma biomarker.

**Materials and methods:**

Quantitative real-time PCR was adopted to detect lnc-MyD88 expression in plasma samples of 98 HCC patients, 52 liver cirrhosis (LC) patients, and 105 healthy people. The correlation between lnc-MyD88 and clinicopathological factors was analyzed through chi-square test. The receiver operating characteristic (ROC) curve was used to analyze the sensitivity, specificity, Youden index, and area under the curve (AUC) of lnc-MyD88 and AFP alone and in combination for the diagnosis of HCC. The relationship between MyD88 and immune infiltration was analyzed by single sample gene set enrichment analysis (ssGSEA) algorithm.

**Results:**

Lnc-MyD88 was highly expressed in plasma samples of HCC and hepatitis B virus (HBV)-associated HCC patients. Lnc-MyD88 had better diagnostic value than AFP in HCC patients using healthy people or LC patients as control (healthy people, AUC: 0.776 vs. 0.725; LC patients, AUC: 0.753 vs. 0.727). The multivariate analysis showed that lnc-MyD88 had great diagnostic value for distinguishing HCC from LC and healthy people. Lnc-MyD88 had no correlation with AFP. Lnc-MyD88 and AFP were independent diagnostic factors for HBV-associated HCC. The AUC, sensitivity, and Youden index of the combined diagnosis of lnc-MyD88 and AFP combined were higher than those of lnc-MyD88 and AFP alone. The ROC curve of lnc-MyD88 for the diagnosis of AFP-negative HCC was plotted with a sensitivity of 80.95%, a specificity of 79.59%, and an AUC value of 0.812 using healthy people as control. The ROC curve also presented its great diagnostic value using LC patients as control (sensitivity: 76.19%, specificity: 69.05%, AUC value: 0.769). Lnc-MyD88 expression was correlated with microvascular invasion in HBV-associated HCC patients. MyD88 was positively correlated with infiltrating immune cells and immune-related genes.

**Conclusion:**

The high expression of plasma lnc-MyD88 in HCC is distinct and could be utilized as a promising diagnostic biomarker. Lnc-MyD88 had great diagnostic value for HBV-associated HCC and AFP-negative HCC, and it had higher efficacy in combination with AFP.

## Introduction

Hepatocellular carcinoma (HCC) is ranked as the sixth most common cancer and also ranked third on the leading causes of cancer-induced mortality across the globe (1). What is more striking is that HCC is also ranked sixth in the malignant tumor incidence and second in mortality in China (2). HCC is one of the common malignancies of the digestive tract, displaying no symptoms in the early stage of the disease in some patients and nonspecific symptoms in others including abdominal pain, fever, and lethargy (3, 4). The clinical manifestations intensify toward the late stage of the disease. Although progress has been made in diagnosing and treating HCC, the prognosis of patients is still very poor due to the delayed diagnosis, limited treatment options, recurrence, and metastasis after surgery (5, 6).

At present, the diagnosis of HCC mainly depends on alpha-fetoprotein (AFP) and imaging technology, but in the early diagnosis of HCC, the missed diagnosis rate (AFP <20 ng/ml) is as high as 30%–40% (7). Even in patients with advanced HCC, 30% of plasma AFP is at a normal level; this type of HCC is also called AFP-negative HCC (AFP-NHCC) (8). Since AFP-NHCC are usually small in size, the rate of diagnosis by B-ultrasound is only approximately 10.4%, and missed diagnosis often occurs when using imaging examinations (9). Therefore, the discovery and application of new plasma markers to improve the ability and efficiency for early diagnosis of HCC have become an urgent need for clinical medicine.

Noncoding RNAs (ncRNAs) have been attracting the attention of many scholars in recent years. A large amount of research data indicate that ncRNAs partake in cell proliferation, differentiation, apoptosis, migration, and invasion (10–12). NcRNAs have been discovered to be of significant importance in cancer research. As a member of ncRNAs, long noncoding RNAs (lncRNAs) are a type of RNA transcript with more than 200 nucleotides long but do not bear any protein-coding potential. Past evidence suggested that lncRNAs are essential in many biological processes, such as transcriptional regulation, cancer progression, and cell differentiation (13, 14). More and more research advocates that lncRNAs are involved in regulating the occurrence and development of cancer. For example, lncRNA ARLNC1 interacts with mRNA encoding the androgen receptor (AR) to induce oncogenic AR signal transduction, proliferation, and survival; it further participates in the occurrence and progression of prostate cancer (15). LncRNA MEG3 can inhibit the rapid increase of Non-small cell lung carcinoma (NSCLC) and induce apoptosis by disturbing the p53 expression (16).

Because lncRNA has a regulatory role, it may have significant implications in a variety of diseases through complex mechanisms. Therefore, looking for lncRNAs related to HCC may be very important for discovering and treating HCC. In addition, past literature has elucidated that certain lncRNAs are associated with the occurrence and development of cancer and can be detected in plasma (17). For example, the reduction of X91348 in plasma could be the diagnostic and prognostic indicators for HCC (18). Therefore, plasma lncRNAs could be utilized as possible biomarkers for early diagnosis, treatment, tumor grading, screening, and prognostic analysis of HCC.

Myeloid differentiation primary response protein 88 (MyD88) was defined as a carcinogenic gene that promoted the occurrence and development of HCC *via* Toll-like receptor 4 (TLR4)-MyD88-Nuclear Factor Kappa-beta (NF-κB) pathway. MyD88 had been considered as a treatment and prognostic evaluation target for HCC (19–22). Previous research has established that lnc-MyD88 increases the expression of MyD88 through enhanced H3K27Ac in the promoter of the MyD88 gene, resulting in the activation of the NF-κB and The phosphatidylinositol 3-kinase/Protein kinase B(AKT) (PI3K/AKT) signaling pathways and then promoting the proliferation and transfer of HCC (23). In the present study, the expression of lnc-MyD88 was detected in HCC plasma specimens *via* qRT-PCR analysis. At the same time, the diagnostic value of lnc-MyD88 was evaluated in the hope of providing evidence for novel biomarkers for early detection of HCC.

## Materials and methods

### The collection of blood samples

The present research was conducted in collaboration with the Affiliated Changzhou No. 2 People’s Hospital of Nanjing Medical University and Changzhou No. 3 People’s Hospital. In total, plasma samples of 98 patients with HCC, 52 patients with liver cirrhosis (LC), and 105 healthy subjects were acquired from the aforementioned hospitals. Prior to surgery, no treatment including chemotherapy, radiotherapy, and antitumor treatment using biological products was performed on patients. According to relevant guidelines, the diagnosis of LC or HCC is based on typical morphological findings and pathological observations using CT or ultrasound (24, 25). Tumor staging and grading for each patient were established using the American joint committee on cancer (AJCC) TNM staging system (Edge & Compton, 2010). The study was approved by the Ethical Committee of the Affiliated Changzhou No. 2 People’s Hospital of Nanjing Medical University, and informed consent of all participants was obtained.

### The preparation of blood samples

Venous blood samples were collected from participants within 2 h, and they were centrifuged at 4,000 rpm for 10 min; this was to detect the level of plasma lnc-MyD88. The supernatant plasma was centrifuged at 12,000 rpm for a further 15 min to eliminate any leftover debris. To avoid hemolysis, the entire procedure was meticulously monitored and controlled, and the plasma was kept at −80°C until RNA was extracted.

### RNA isolation and qRT-PCR analysis

Total RNA extraction was achieved through the TRIzol LS reagent (Invitrogen, Carlsbad, CA, USA) (Lot no. 223306) following the guide supplied by the manufacturer. NanoDrop spectrophotometer was used to assess the quality and quantify RNA. The reverse transcription (RT) reactions took place using the Prime Script™ RT Reagent Kit (Takara, Dalian, Liaoning) (Lot no. AK4802). The 20-μl solution contained five samples of 4-μl Prime Script Buffer Mix, 1 μg of template RNA and 1 μg of Prime Script RT Enzyme Mix I, 1 μl of Oligo dT Primer and RNase-free H2O; it was then incubated firstly at 37°C for 30 min, followed by 85°C for 5 s, and finally 4°C for 60 min. RT-qPCR was completed using a CFX96™ real-time system (BioRad, CA, USA). The cDNA solution generated was diluted, and 2 μl of this solution was then combined with 12.5 μl of SYBR Premix Ex TaqTM, 0.5 μl of Dye II (Vazyme Biotech, Nanjing, China) (Lot no. 7E480I0), 1 μl forward and reverse primers (10 μM), and 9 μl of nuclease-free water, so that the total volume was 25 μl following the guideline by the manufacturer (Takara, Dalian, China). Then, the solution was incubated at 95°C for 30 s, after that, 45 cycles of 95°C and 60°C alternating for 5 s and 34 s, respectively. Three repetitions were the minimum standard for each test. The specificity of the RT-qPCR products was examined with the melting curve analysis. The relative gene expression level was normalized by the endogenous control-β-actin, then assessed using the 2^−ΔΔCt^ method. The primer sequences were synthesized by RiboBio (Guangzhou, China). Lnc-MyD88 primer sequences: forward, 5′-TACCACAGGGGCTGGAGTTAT-3′; reverse, 5′-AGTCTGTCCCCCTCCAGGTT-3′; β-actin primer sequences: forward, 5′-CGGAGTCAACGGATTTGGTCGTATTGG-3′; reverse, 5′-CCATGGTGTCTGAGCGATGT-3′.

### Statistical analysis

The results were illustrated as a ranking of means yielded or percentages. Among the three groups of clinical data, one-way ANOVA was used for measurement data, and chi-square (x^2^) tests were used for count data. Nonparametric tests including Mann–Whitney and Kruskal–Wallis were also utilized to contrast skewed RNA data (2^−ΔΔCt^) from independent samples. The diagnostic accuracies of the parameters examined were assessed by the receiver operating characteristic (ROC) curve analysis. In addition, the logistic regression analysis was performed in order to recognize the predictor RNA that is correlated with the predisposition of developing HCC. The association between the expression of individual RNA subtype and the occurrence of HCC was established by conducting a univariate analysis. Spearman rank correlation test determined the relationship between the two quantitative nonparametric variables. Data input and statistical analyses were performed using the Statistical Package for the Social Science (SPSS, Chicago, IL, USA) version 22.0 software. Significance level was set at the *P* < 0.05 with a 95% confidence interval (CI). The association between MyD88 and immune cells was analyzed through the ssGSEA algorithm of Gene Set Variation Analysis (GSVA) package (1.34.0) in R software (3.6.3). The immune cells incorporated cytotoxic cells, eosinophils, dendritic cells (DCs), immature DCs (iDCs), activated DCs (aDC), CD8 T cells, B cells, natural killer (NK) CD56bright cells, neutrophils, NK cells, NK CD56dim cells, mast cells, macrophages, T cells, plasmacytoid DCs (pDCs), T helper cells, T effector memory (Tem), T follicular helper (Tfh), T central memory (Tcm), T gamma delta (Tgd), Th1 cells, Th2 cells, Th17 cells, and Treg25. The correlation in respect of the immune-related gene expression and MyD88 expression was analyzed and visualized by employing R software (3.6.3) and ggplot2 (3.3.3) package.

## Results

### Demographics, laboratory investigations, and clinical features of the patients

A total of 255 individuals participated in this present study, including 98 HCC patients (84 men and 14 women), 52 patients with LC (38 men and 14 women), and 105 healthy subjects (40 men and 65 women). In the LC group, 67.3% of the patients were classified as Child A, 26.9% were classified as Child B, and 5.8% were classified as Child C. Whereas none was scored as Child C, 96.9% were scored as Child A, and 3.1% were scored as Child B (*P* < 0.05) among HCC patients. By comparing the clinicopathological data of 255 individuals in the three groups, we found that the subjects in the three groups had significant differences in Hepatitis B surface antigen (HBsAg), Hepatitis B e-antigen (HBeAg), and Child grades (*P* < 0.05; **Table 1**). In HCC patients, 94.9% had early and middle Barcelona Clinic Liver Cancer (BCLC) stage (BCLC stage 0+A+B), and 5.1% of patients had advanced BCLC stage (BCLC stage C). The TNM stage was IA in 24.5% of the patients, and the TNM stage in 34.7% of the patients was stage IB. In addition, 30.6% of the patients had stage II, and 5.1% of the patients had stage IIIA (**Table 2**). Lastly, 5.1% of the patients had stage IIIB. The tumor volume of 34.7% of the patients was smaller than 3 cm (**Table 2**). The tumor volume of 65.3% of the patients was larger than 3 cm (**Table 2**). Clinically healthy gender-matched individuals with similar ages (n = 105) served as a control group. Results indicated a major statistically significant difference between HCC patients, LC patients, and healthy subjects with regard to the mean values ± SD of platelet (PLT) count, hemoglobin (HGB), PLT, international normalized ratio (INR), fibrinogen (FBG), alanine aminotransferase (ALT), alkaline phosphatase (ALP), total bilirubin (TBIL), direct bilirubin (DBIL), albumin (ALB), AFP, carcinoembryonic antigen (CEA), and carbohydrate antigen 125 (CA125) (*P* > 0.05; **Table 3**). A total of 82 hepatitis B virus (HBV)-associated HCC cases were screened out from 98 HCC patients, 48 HBV-associated LC cases were screened out from 52 LC patients, and 53 healthy subjects who were treated during the same period were used as controls. There were no statistically significant differences in the general clinical data such as age, gender, and Body Mass lndex (BMI) among the healthy control group, HBV-associated LC group, and HBV-associated HCC group (*P* > 0.05; **Table 4**).

**Table 1:** Demographic, clinical, and some biochemical characteristics of the study groups.

	Normal (n=105)	LC (n=52)	HCC (n=98)	*P*	χ^2^
Age				*0.067*	χ^2^ *=5.405*
<60 years (n=114)	40 (38.1%)	30 (57.7%)	44 (44.9%)		
≥60 years (n=141)	65 (61.9%)	22 (42.3%)	54 (55.1%)		
Gender				*0.681*	χ^2^ *=0.767*
Male (n=162)	62 (59.0%)	28 (53.8%)	60 (61.2%)		
Female (n=93)	43 (41.0%)	24 (46.2%)	38 (38.8%)		
BMI				*0.314*	χ^2^ *=2.317*
<25 (n=150)	79 (75.2%)	43 (82.7%)	70 (71.4%)		
≥25 (n=105)	26 (24.8%)	9 (17.3%)	28 (28.6%)		
HBsAg				<0.0001	χ^2^=182.663
Positive (n=131)	1 (1%)	48 (92.3%)	82 (83.7%)		
Negative (n=124)	104 (99%)	4 (7.7%)	16 (16.3%)		
HBeAg				*0.021*	χ^2^ *=7.766*
Positive (n=11)	1 (1%)	2 (3.8%)	9 (9.2%)		
Negative (n=244)	104 (99%)	50 (96.2%)	89 (90.8%)		
Child–Pugh Grade				*<0.0001*	χ^2^ *=26.034*
A		35 (67.3%)	95 (96.9%)		
B		14 (26.9%)	3 (3.1%)		
C		3 (5.8%)	0 (0%)		

Data are shown as number (percentage) or mean ± standard deviation. Chi-square, Kruskal–Wallis, and ANOVA tests were used followed by Tukey multiple comparison test.

LC, liver cirrhosis; HCC, Hepatocellular carcinoma; BMI, Body Mass lndex; HBsAg, Hepatitis B surface antigen; HbeAg, Hepatitis B e-antigen.

**Table 2:** The clinicopathological parameters of HCC patients.

	HCC (n=98)
Tumor stage (BCLC)	
A+B	93 (94.9%)
C	5 (5.1%)
Tumor stage (TNM)	
IA	24 (24.5%)
IB	34 (34.7%)
II	30 (30.6%)
IIIA	5 (5.1%)
IIIB	5 (5.1%)
Tumor diameter	
<3 cm	34 (34.7%)
≥3 cm	64 (65.3%)

HCC, Hepatocellular carcinoma; BCLC, Barcelona Clinic Liver Cancer.

**Table 3:** Demographic and laboratory data of all studied groups.

	Normal (n=105)	LC (n=52)	HCC (n=98)	*P*
Age	54.8±15.05	57.98±11.35	56.3±9.76	0.3138
BMI	23.24±3.312	22.94±3.011	23.49±3.157	0.5954
HGB (g/L)	131±19.38	127.7±24.21	145.6±17.29	<0.0001*
PLT*10^9^/L	224.4±75.66	129.3±82.46	151.3±67.87	<0.0001*
INR	0.973±0.08	1.175±0.221	1.023±0.074	<0.0001*
FBG (g/L)	3.046±1.143	2.421±0.892	2.825±0.778	0.0009*
ALT (U/L)	18.4 (12.1,29.2)	29.5 (19.3,43.0)	34.5 (21.8,55.0)	<0.0001*
AST (U/L)	20.5 (16.3,27.3)	34.5 (22.0,59.5)	28.0 (20.0,39.0)	0.1052
ALP (U/L)	70 (56,85)	103 (75,143)	90 (77,118)	<0.0001*
GGT (U/L)	23 (13,51)	51 (29,111)	52 (31,97)	0.6026
TBIL (μmol/L)	16.01±16.23	35.87±51.14	19.19±17.51	0.0001*
DBIL (μmol/L)	7.527±11.83	17.5±34.39	6.767±8.971	0.0013*
TP (g/L)	67.59±7.284	66.9±7.765	68.95±5.669	0.1656
ALB (g/L)	42.8±5.238	36.23±6.506	41.75±3.505	<0.0001*
AFP (ng/ml)	3.19 (2.44,4.15)	2.87 (1.90,4.55)	8.80 (2.74,46.65)	0.001*
CEA (ng/ml)	1.44 (0.82,2.07)	2.16 (1.32,3.18)	2.36 (1.53,3.39)	0.0172*
CA19-9 (U/ml)	8.89 (6.00,17.09)	17.45 (7.04,33.45)	15.50 (6.70,31.20)	0.2222
CA125 (U/ml)	9.44 (7.14,13.93)	15.05 (9.20,37.28)	9.95 (6.30,28.88)	0.0004*

*Asterisks indicate that there was a significance difference (P ≤ 0.05).

HGB, hemoglobin; PLT, platelet; INR, international normalized ratio; FBG, fibrinogen; ALT, alanine aminotransferase; AST, aspartate aminotransferase; ALP, alkaline phosphatase; GGT, γ-glutamyl transpeptidase; TBIL, total bilirubin; DBIL, direct bilirubin; TP, total protein; ALB, albumin; AFP, alpha-fetoprotein; CEA, carcinoembryonic antigen; CA19-9, carbohydrate antigen 19-9; CA125, carbohydrate antigen 125; LC, liver cirrhosis; HCC, Hepatocellular carcinoma; BMI, Body Mass lndex.

**Table 4:** The clinical data of patients.

Clinical factors		Healthy control (n=53)	HBV-related liver cirrhosis(n=48)	HBV-related HCC(n=82)	*P* value
Gender	Male	40	35	72	0.069
	Female	13	13	10	
Age		55.21±16.31	57.85±11.66	55.81±9.78	0.534
BMI (kg/m^2^)		23.73±3.50	22.94±3.02	23.46±3.29	0.472
AFP (ng/ml)		3.04 (2.61,4.13)	2.95 (2.07,4.55)	9.65 (3.37,71.54)	0.000
AST (U/L)		21.50 (16.10,30.45)	34.50 (22.00,63.34)	28.00 (20.00,39.00)	0.000
ALT (U/L)		20.80 (13.65,38.50)	30.50 (20.00,43.75)	33.50 (20.75,53.00)	0.004
ALP (U/L)		70.00 (57.00,90.50)	105.00 (74.75,142.75)	87.50 (73.75,115.00)	0.000
GGT (U/L)		32.00 (19.00,69.50)	52.50 (29.00,110.75)	51.50 (29.50, 92.50)	0.024
ALB (g/L)		44.20 (38.45,47.60)	36.35 (30.98,43.10)	42.20 (39.28,44.20)	0.000
TBIL (μmol/L)		11.90 (10.05,17.65)	23.45 (15.10,38.20)	16.35 (12.55,19.30)	0.000
CEA (ng/ml)		1.51 (0.96,3.00)	2.16 (1.35,3.18)	2.31 (1.59,3.37)	0.026
CA19-9 (U/ml)		8.56 (5.41, 14.43)	19.50 (8.75,37.80)	14.65 (6.38, 26.30)	0.002
CA125 (U/ml)		8.75 (6.54,12.17)	14.89 (9.20,33.23)	9.95 (6.30,14.40)	0.002
Child–Pugh	A	–	31	80	0.000
	B+C	–	17	2	

HBV, hepatitis B virus.

### Expression of plasma lnc-MyD88 among the study groups

**Figure 1:**
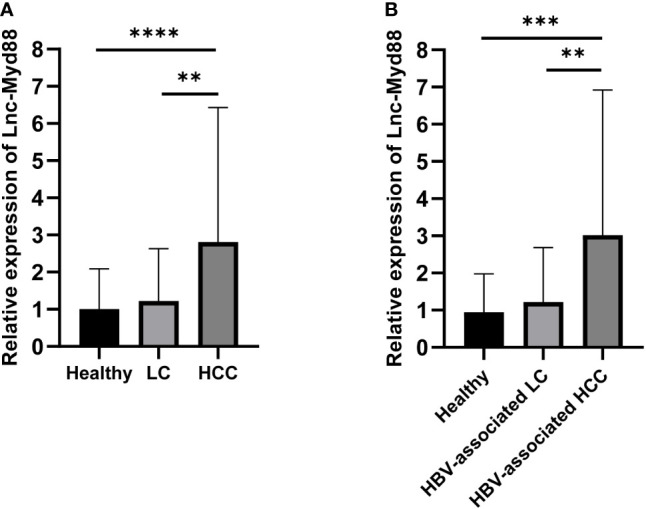
**(A)** Relative expression levels of lnc-MyD88 in HCC patients, LC patients, and normal people. **(B)** Relative expression levels of lnc-MyD88 in HBV-associated HCC patients, HBV-associated LC patients, and normal people. **P < 0.01, ***P < 0.001, ****P < 0.0001. LC, liver cirrhosis; HCC, Hepatocellular carcinoma; HBV, Hepatitis B virus; Lnc-Myd88: Long noncoding RNA-Myd88.

To establish the level of expression of lnc-MyD88 in HCC, LC, and healthy subjects, the plasma levels of lnc-MyD88 were measured in all three groups, and the results illustrated in **Figure 1A** indicated a considerable upregulation of the lnc-MyD88 in patients with HCC compared with healthy subjects (*P* < 0.0001; **Figure 1A**). A similar trend is also observed when compared with patients with LC (*P* < 0.05; **Figure 1A**). The relative expression level of plasma lnc-MyD88 in the HBV-associated HCC group was higher than that in the healthy control group and HBV-associated LC group (*P* < 0.001; **Figure 1B**).

### The diagnostic value of long noncoding RNA-Myd88 in hepatocellular carcinoma

**Figure 2:**
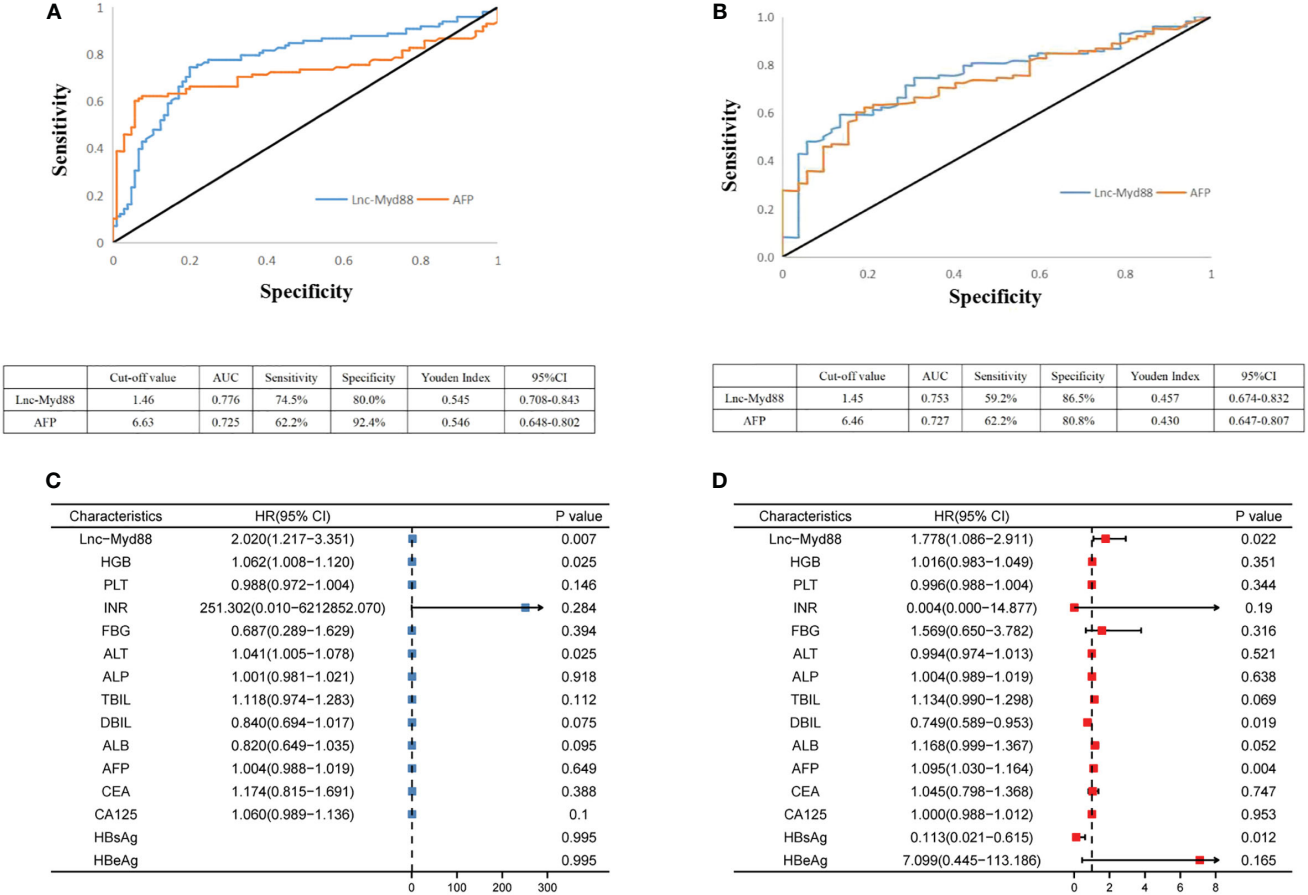
**(A)** ROC curve analysis of lnc-MyD88 and AFP expression in differentiating patients with HCC (n = 98) from healthy people (n = 105). **(B)** ROC curve analysis of lnc-MyD88 and AFP expression in differentiating patients with HCC (n = 98) from patients with LC (n = 52). **(C)** The multivariate analysis result indicated the diagnostic value of lnc-MyD88 for distinguishing healthy individuals from HCC patients. **(D)** The multivariate analysis result indicated the diagnostic value of lnc-MyD88 for distinguishing LC patients from HCC patients. LC, Liver cirrhosis; HCC, Hepatocellular carcinoma; HBV, Hepatitis B virus; Lnc-Myd88, Long noncoding RNA-Myd88; HBsAg, Hepatitis B surface antigen; HbeAg, Hepatitis B e-antigen.

The diagnostic value of lnc-MyD88 and AFP in HCC patients was appraised by the ROC curve analysis. It was discovered that plasma levels (lnc-MyD88 or AFP) could distinguish HCC patients from healthy people, with areas under the curves (AUCs) being 0.776 and 0.725, respectively. The sensitivities were 74.5% and 62.2% at the cutoff values of 1.46 and 6.63, respectively; and the specificities were 80.0% and 92.4% (**Figure 2A**). Comparisons between HCC and LC patients were also made in order to determine whether lnc-MyD88 or AFP aberrant expression was HCC-specific, and the cutoff values were 1.45 and 6.46 for lnc-MyD88 and AFP, respectively; which could be used to distinguish the two groups. The cutoff values gave insight onto the decreased sensitivity (59.2%) and the increased specificity (86.5%) of lnc-MyD88. AFP had decreased specificity (80.8%) but the sensitivity remained the same (**Figure 2B**). Together, lnc-MyD88 and AFP are potentially better diagnostic tools for distinguishing HCC from healthy subjects than the LC group. The result of multivariate analysis showed that lnc-MyD88 [Hazard Ratio (HR) = 2.020, 95% CI = 1.217–3.351, *P=* 0.007], HGB (HR = 1.062, 95% CI = 1.008–1.120, *P =* 0.025), and ALT (HR = 1.041, 95% CI = 1.005–1.078, *P =* 0.025) had potential to distinguish HCC patients from healthy subjects (**Figure 2C**). The expression of lnc-MyD88 (HR = 1.778, 95% CI = 1.086–2.911, *P* = 0.022), DBIL (HR = 0.749, 95% CI = 0.589–0.953, *P =* 0.019), AFP (HR = 1.095, 95% CI = 1.030–1.164, *P =* 0.004), and HBsAg (HR = 0.113, 95% CI = 0.021–0.615, *P =* 0.012) had potential to distinguish HCC patients from LC patients (**Figure 2D**).

### The correlation analysis between lnc-MyD88 expression and laboratory investigations

The expression level of lnc-MyD88 in the plasma of HCC patients was detected by qRT-PCR, and various laboratory investigations were collected from the clinical data. The Spearman analysis was conducted, and the result showed that there is no significant difference between lnc-MyD88 expression and various laboratory investigations including AFP (**Table 5**). However, the expression level of AFP is positively correlated with the level of aspartate aminotransferase (AST) in plasma (Cor = 0.281, *P =* 0.031; **Table 5**).

**Table 5:** The correlation analysis of lnc-MyD88 and other testing indexes.

			Lnc-MyD88	AFP	Age	AST	ALT	TBIL	DBIL	ALB	INR
Spearman	Lnc-MyD88	Cor	1.000	0.009	-0.091	-0.118	-0.01 9	-0.016	-0.054	0.18 7	0.187
		*P*		0.930	0.373	0.248	0.852	0.876	0.598	0.06 5	0.065
		n	98	98	98	98	98	98	98	98	98
	AFP	Cor	0.009	1.000	-0.177	0.281	0.164	-0.059	-0.012	0.00 1	0.001
		*P*	0.930		0.081	**0.031**	0.107	0.563	0.903	0.98 9	0.989
		n	98	98	98	98	98	98	98	98	98

Bold value were represents the value is less than 0.05.

Lnc-Myd88, Long noncoding RNA-Myd88.

### The combined diagnostic value of lnc-MyD88 and AFP in HBV-associated HCC

**Figure 3:**
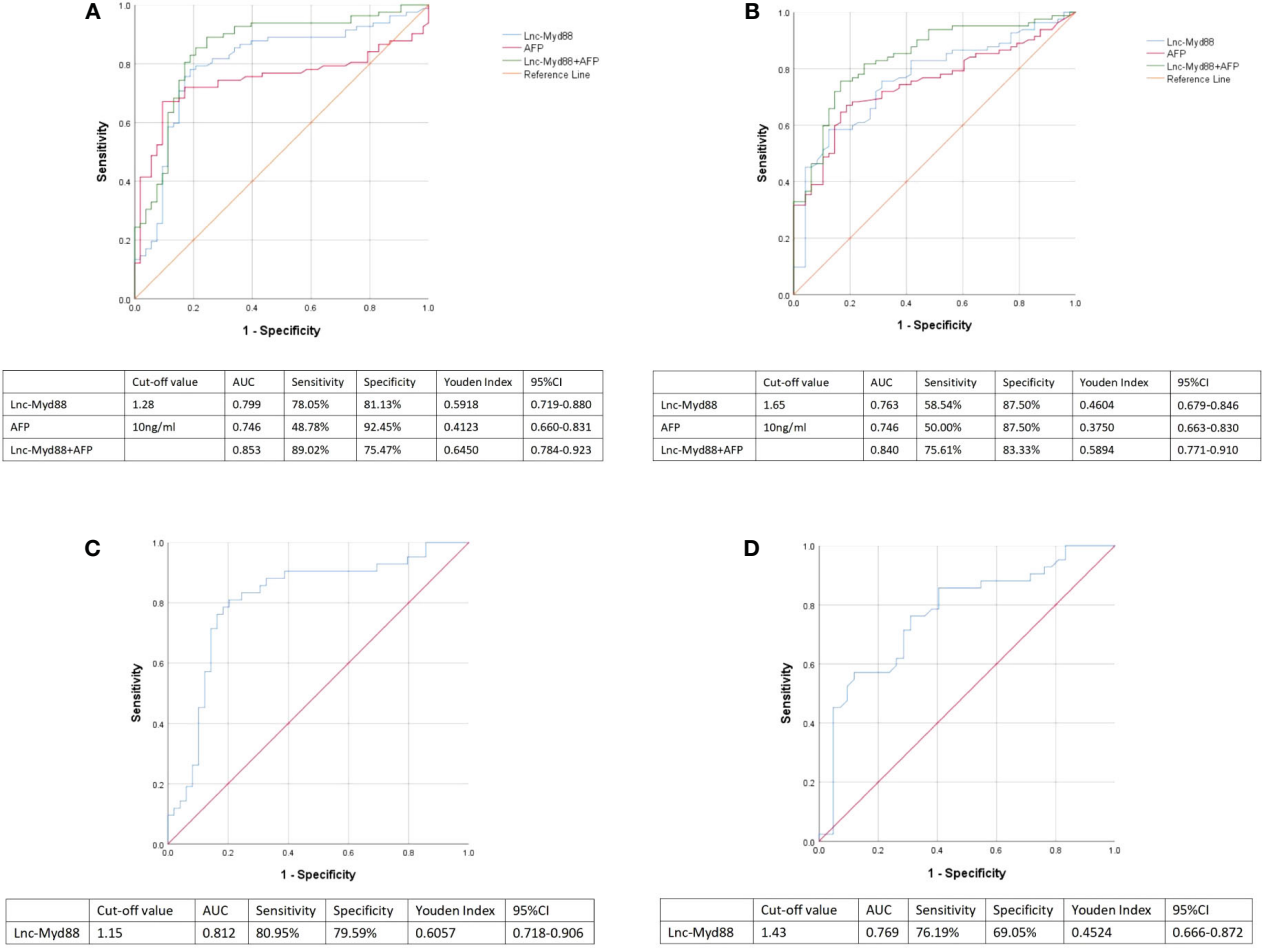
**(A)** ROC curves were used to analyze the sensitivity, specificity, Youden index, and area under the curve (AUC) of lnc-MyD88 and AFP alone and in combination for the diagnosis of HBV-associated HCC. Healthy people were set as control. **(B)** ROC curves were used to analyze the sensitivity, specificity, Youden index, and AUC of lnc-MyD88 and AFP alone and in combination for the diagnosis of HBV-associated HCC. LC patients were set as control. **(C)** The ROC curve of plasma lnc-MyD88 for the diagnosis of AFP-negative HCC was plotted using healthy people as the control group. **(D)** The ROC curve of plasma lnc-MyD88 for the diagnosis of AFP-negative HCC was plotted using LC patients as the control group. LC, Liver cirrhosis; HCC: Hepatocellular carcinoma; HBV, Hepatitis B virus; Lnc-Myd88, Long noncoding RNA-Myd88.

Logistic regression analysis was used to explore the independent risk factors for HBV-associated HCC. When we set healthy people as the control group, univariate analysis results indicated that lnc-MyD88, AFP, ALT, ALP, and CEA were risk factors of HBV-associated HCC (**Table 6**). Multivariate analysis showed that lnc-MyD88 and AFP were independent risk factors of HBV-associated HCC (**Table 6**). Compared with patients with HBV-associated LC, lnc-MyD88, AFP, AST, ALP, ALB, TBIL, carbohydrate antigen 19-9 (CA19-9), and CA125 were risk factors for HBV-associated HCC in the univariate analysis (**Table 7**), and lnc-MyD88, AFP, and ALB were independent risk factors for HBV-associated HCC (**Table 7**). The ROC curve was drawn with the healthy physical examination group as the control, and the results showed that the AUC of lnc-MyD88 for diagnosing HBV-associated HCC was 0.799 (**Figure 3A**), which was slightly higher than that of AFP, which was 0.746 (**Figure 3A**). The AUC of the combined diagnosis of lnc-MyD88 and AFP was the largest, which was 0.853 (**Figure 3A**), higher than that of lnc-MyD88 and AFP alone. Among them, the combined detection of lnc-MyD88 and AFP had the highest sensitivity and Youden index (**Figure 3A**). The ROC curve was drawn with the HBV-associated LC group as the control, and the results showed that the AUC of lnc-MyD88 for diagnosing HBV-associated HCC was 0.763 (**Figure 3B**), which was slightly higher than that of AFP, which was 0.746 (**Figure 3B**). The AUC of the combined diagnosis of lnc-MyD88 and AFP was the largest, which was 0.840 (**Figure 3B**), higher than that of lnc-MyD88 and AFP alone. Among them, the combined detection of lnc-MyD88 and AFP had the highest sensitivity and Youden index (**Figure 3B**).

**Table 6:** The univariate and multivariate analyses indicated the diagnostic value of various clinical factors (including lnc-MyD88 and AFP) in HBV-related HCC group, and the healthy people group is the control.

Factors	Univariate analysis		Multivariate analysis	
	OR (95% *CI*)	*P* value	OR (95% *CI*)	*P* value
Age	1.004 (0.977~1.032)	0.785		
BMI (kg/m^2^)	0.977 (0.881~1.082)	0.650		
Lnc-MyD88	2.462 (1.575~3.849)	**0.000**	2.582 (1.623~4.109)	0.000
AFP (ng/ml)	1.017 (1.001~1.034)	**0.043**	1.014 (1.001~1.027)	0.037
AST (U/L)	0.997 (0.989~1.005)	0.459		
ALT (U/L)	1.023 (1.006~1.014)	**0.007**	1.019 (1.000~1.038)	0.052
ALP (U/L)	1.010 (1.000~1.021)	0.059	1.006 (0.097~1.015)	0.216
GGT (U/L)	1.000 (0.997~1.003)	0.851		
ALB (g/L)	0.967 (0.893~1.047)	0.410		
TBIL (μmol/L)	1.004 (0.982~1.026)	0.745		
CEA (ng/ml)	1.231 (0.981~1.546)	0.073	1.206 (0.941~1.544)	0.138
CA19-9 (U/ml)	1.000 (0.991~1.009)	0.957		
CA125 (U/ml)	1.013 (0.993~1.033)	0.200		

Bold value were represents the value is less than 0.05.

Lnc-Myd88, Long noncoding RNA-Myd88; HBV, hepatitis B virus; BMI, Body Mass lndex.

**Table 7:** The univariate and multivariate analyses indicated the diagnostic value of various clinical factors (including lnc-MyD88 and AFP) in HBV-related HCC group, and the HBV-related liver cirrhosis group is the control.

Factors	Univariate analysis		Multivariate analysis	
	OR (95% *CI*)	*P* value	OR (95% *CI*)	*P* value
Age	0.981 (0.948~1.016)	0.286		
BMI (kg/m^2^)	1.054 (0.940~1.183)	0.368		
Lnc-MyD88	1.694 (1.160~2.473)	**0.006**	1.543 (1.066~2.233)	**0.021**
AFP (ng/ml)	1.054 (1.014~1.096)	**0.008**	1.073 (1.015~1.134)	**0.014**
AST (U/L)	0.982 (0.969~0.995)	**0.007**	0.986 (0.964~1.009)	0.227
ALT (U/L)	1.004 (0.992~1.017)	0.501		
ALP (U/L)	0.991 (0.983~0.998)	**0.018**	0.999 (0.987~1.010)	0.832
GGT (U/L)	0.999 (0.995~1.002)	0.537		
ALB (g/L)	1.242 (1.134~1.360)	**0.000**	1.198 (1.065~1.348)	**0.003**
TBIL (μmol/L)	0.963 (0.936~0.991)	**0.010**	0.999 (0.967~1.032)	0.954
CEA (ng/ml)	1.068 (0.905~1.260)	0.435		
CA19-9 (U/ml)	0.984 (0967~1.002)	0.080	0.995 (0.970~1.020)	0.689
CA125 (U/ml)	0.993 (0.986~0.999)	**0.034**	0.996 (0.986~1.006)	0.451

Bold value were represents the value is less than 0.05.

### The diagnostic value of lnc-MyD88 in AFP-negative HCC

The ROC curve of plasma lnc-MyD88 for the diagnosis of AFP-negative HCC was plotted with a sensitivity of 80.95%, a specificity of 79.59%, and an AUC value of 0.812 using healthy people as the control group (**Figure 3C**). The ROC curve of plasma lnc-MyD88 for the diagnosis of AFP-negative HCC was plotted with a sensitivity of 76.19%, a specificity of 69.05%, an AUC value of 0.769, and the LC people were used as the control group (**Figure 3D**).

### The presentation of lnc-MyD88 functions in different clinicopathological data

In order to further analyze the correlation between lnc-MyD88 expression and various clinicopathological data, we divided HCC patients into high lnc-MyD88 expressed group (n = 49) and low lnc-MyD88 expressed group (n = 49) based on the median value. The result showed that the expression of lnc-MyD88 in the plasma of HCC patients has no significant correlation with various clinicopathological data (*P* > 0.05; **Table 8**). In patients with HBV-associated HCC, statistical analysis showed a correlation between high plasma lnc-MyD88 expression and the presence of microvascular invasion (*P* < 0.05; **Table 9**), but no significant correlation of lnc-MyD88 and other clinical parameters was found.

**Table 8:** The association between the lnc-MyD88 expression levels and various clinicopathological features in HCC.

Characteristics	High lnc-MyD88 expression (n=49)	Low lnc-MyD88 expression (n=49)	*P* value
Age			1.000
<60	27	27	
≥60	22	22	
Gender			0.564
Male	43	41	
Female	6	8	
BMI			0.831
<25	33	32	
≥25	16	17	
HBsAg			0.274
Positive	39	43	
Negative	10	6	
HBeAg			0.812
Positive	4	5	
Negative	45	44	
Child–Pugh grade			0.558
A	47	48	
B	2	1	
C	0	0	
BCLC stage			0.646
A+B	46	47	
C	3	2	
TNM stage			0.320
IA	9	15	
IB	20	14	
II	14	16	
IIIA	2	3	
IIIB	4	1	
Tumor Diameter			0.396
<3	15	19	
≥3	34	30	
AFP			0.835
<20	31	30	
≥20	18	19	

Lnc-Myd88, Long noncoding RNA-Myd88; BMI, Body Mass lndex; HBsAg, Hepatitis B surface antigen; HbeAg, Hepatitis B e-antigen; BCLC, Barcelona Clinic Liver Cancer.

**Table 9:** The correlation of lnc-MyD88 expression and various clinicopathological characteristics in HBV-related HCC.

Factors	Number	Low lnc-MyD88 expression	High lnc-MyD88 expression	χ²	*P* value
Total	82	41	41		
Age				0.210	0.647
≤60	52	27	25		
>60	30	14	16		
Gender				0.000	1.000
Male	72	36	36		
Female	10	5	5		
Tumor diameter				0.456	0.499
≤3cm	49	26	23		
>3cm	33	15	18		
Tumor number				0.000	1.000
1	70	35	35		
>1	12	6	6		
Tumor differentiation				0.205	0.651
Medium–High differentiation	53	28	25		
Low differentiation	29	13	16		
Vascular invasion				4.038	0.044
Yes	35	13	22		
No	47	28	19		
Child–Pugh Grade					1.000 (Fisher)
A	80	40	40		
B	2	1	1		
BCLC Stage				0.1566	0.693
0+A	75	37	38		
B+C	7	4	3		
TNM Stage				1.775	0.412
I stage	42	24	18		
II stage	31	13	18		
III stage	9	4	5		

Lnc-Myd88, Long noncoding RNA-Myd88; BCLC, Barcelona Clinic Liver Cancer.

### The strong relationship of MyD88 and infiltrating immune cells

**Figure 4:**
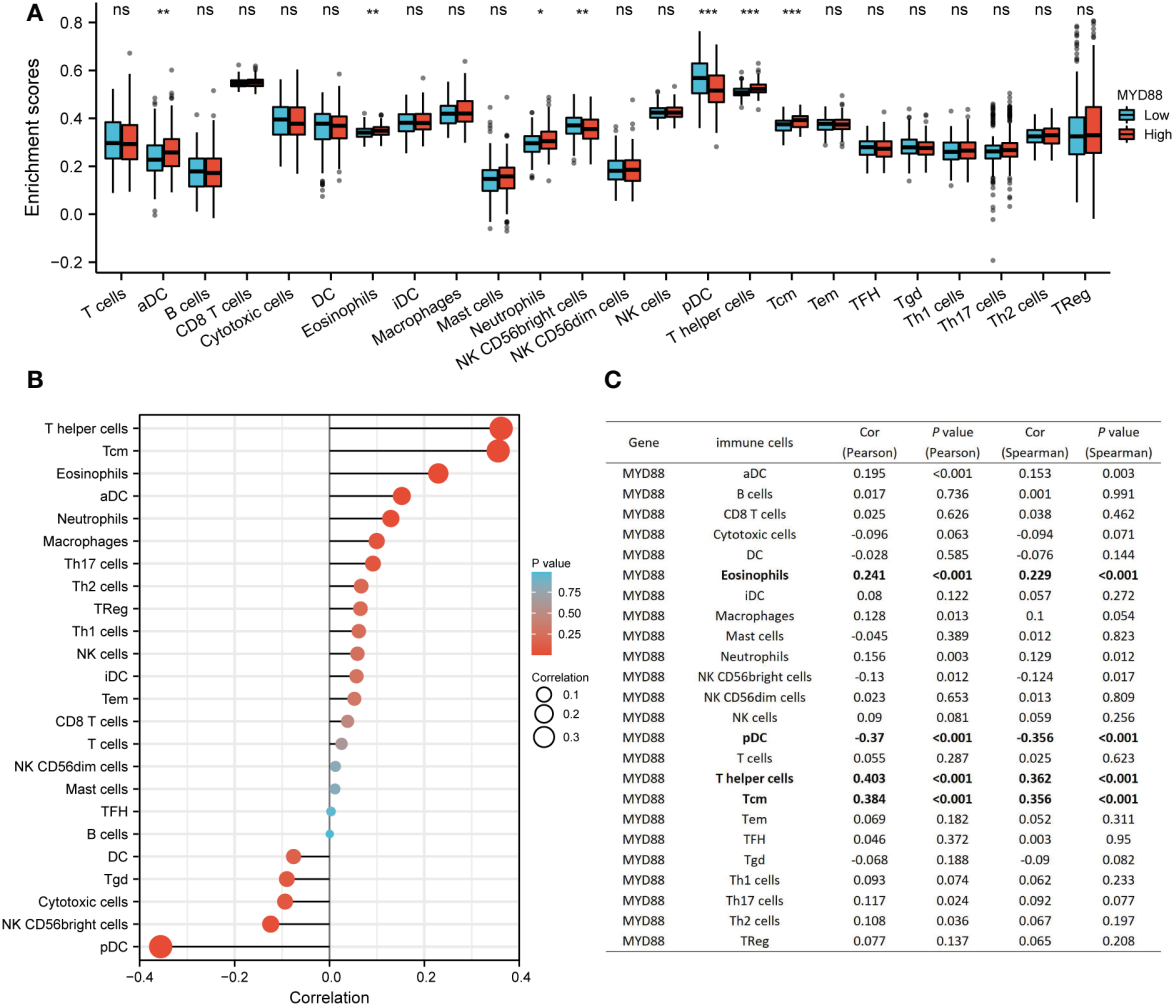
The relationship between MyD88 and immune cells. **(A)** The differential infiltration level of immune cells between the high MyD88 group and the low MyD88 group. *P < 0.05, **P < 0.01, ***P < 0.01. **(B, C)** The Spearman correlation analysis between MyD88 and immune cells was estimated through the ssGSEA algorithm. Myd88, Myeloid differentiation primary response protein 88; ssGSEA, Single sample gene set enrichment analysis; DC, dendritic cells; NK, natural killer.

Our previous research determined that lnc-MyD88 expression had a positive correlation with MyD88 expression by utilizing Pearson analysis (N = 110, r^2^ = 0.5665, *P* < 0.001) (23). Emerging evidence identified the immune-related mechanism involved in the carcinogenic process of HCC (26). The relationship between MyD88 and infiltrating immune cells was explored. The results indicated that aDC, eosinophils, neutrophils, T helper cells, and Tcm were highly infiltrated in the high MyD88-expressed cohort (*P* < 0.05; **Figure 4A**). NK CD56bright cells and pDCs were highly infiltrated in the low MyD88 expression cohort (*P* < 0.05; **Figure 4A**). MyD88 had a positive correlation with eosinophils (Pearson: cor = 0.241, *P* < 0.001; Spearman: cor = 0.229, *P* < 0.001; **Figures 4B, C** **)**, T helper cells (Pearson: cor = 0.403, *P* < 0.001; Spearman: cor = 0.362, *P* < 0.001; **Figures 4B, C** **)**, and Tcm (Pearson: cor = 0.384, *P* < 0.001; Spearman: cor = 0.356, *P* < 0.001; **Figures 4B, C** **)**. MyD88 had a negative correlation with pDCs (Pearson: cor = -0.37, *P* < 0.001; Spearman: cor = -0.356, *P* < 0.001; **Figures 4B, C** **)**.

### MyD88 was correlated with immune-related genes

**Figure 5:**
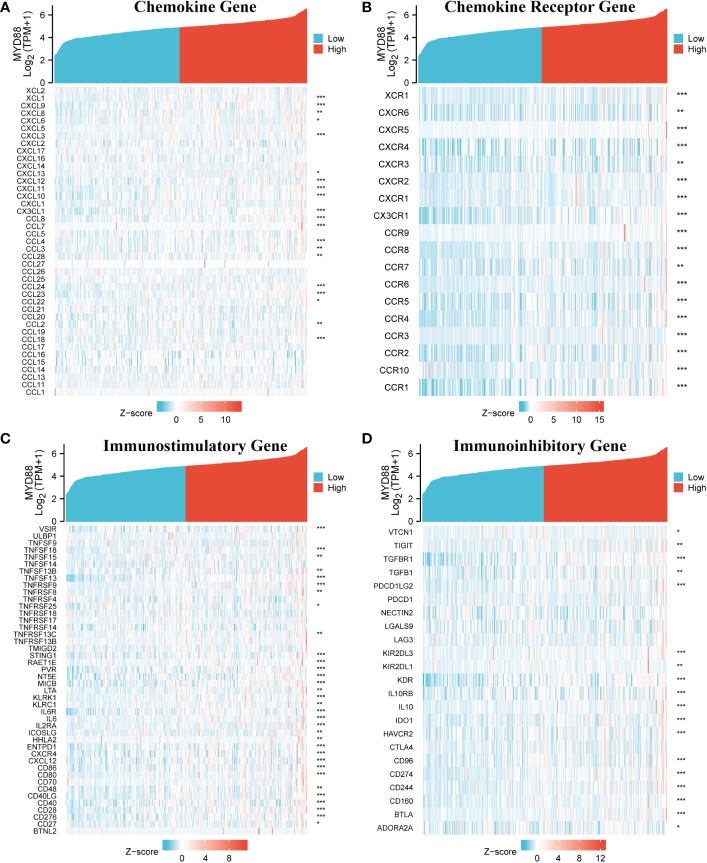
The relationship between MyD88 and immune-related genes. **(A)** The relationship between MyD88 and chemokine genes. **(B)** The relationship between MyD88 and chemokine receptor genes. **(C)** The relationship between MyD88 and immunostimulatory genes. **(D)** The relationship between MyD88 and immunoinhibitory genes. *P < 0.05, **P < 0.01, ***P < 0.001.

The Cancer Genome Atlas-Liver hepatocellular carcinoma (TCGA-LIHC) RNA sequencing data were downloaded from TCGA online platform, and the immune-related analysis was conducted by R software. The outcome indicated that MyD88 expression was related to several chemokine genes including *XCL1, CXCL9, CXCL8, CXCL6 CXCL3, CXCL13, CXCL12, CXCL11, CXCL10, CX3CL1, CCL8, CCL7, CCL4, CCL3, CCL28, CCL24, CCL23, CCL22, CCL2, *and* CCL18* (*P* < 0.05, **Figure 5A**). The chemokine receptor genes (*XCR1, CXCR6, CXCR5, CXCR4, CXCR3, CXCR2, CXCR1, CX3CR1, CCR9, CCR8, CCR7, CCR6, CCR5, CCR4, CCR3, CCR2, CCR10 *and* CCR1*) were positively correlated with the expression of MyD88 (*P* < 0.05, **Figure 5B**). The immunostimulatory genes (*VSIR, TNFSF18, TNFSF15, TNFSF13B, TNFSF13, TNFRSF9, TNFRSF8, TNFRSF25, TNFRSF13C, STING1, RAET1E, PVR, NT5E, MICB, LTA, KLRK1, KLRC1, IL6R, IL6, IL2RA, ICOSLG, HHLA2, ENTPD1, CXCR4, CXCL12, CD86, CD80, CD48, CD40LG, CD40, CD28, CD276, *and* CD27*) were all associated with MyD88 expression (*P* < 0.05; **Figure 5C**). The expression of MyD88 was positively correlated with the immunoinhibitory genes (*VTCN1, TIGIT, TGFBR1, TGFB1, PDCD1LG2, KIR2DL3, KIR2DL1, KDR, IL10RB, IL10, IDO1, HAVCR2, CD96, CD274, CD244, CD160, BTLA *and* ADORA2A*; *P* < 0.05; **Figure 5D**).

## Discussion

HCC is a highly invasive, highly metastatic cancer, and it has a high rate of recurrence and low rate of survival (27). The main reason behind the poor prognosis of HCC is the low rate of early diagnosis. Although tumor markers such as AFP have been developed for the diagnosis of HCC, which has effectively improved the early diagnosis rate, there are still some patients who are missed (28). Therefore, it is essential to find a biomarker for HCC with higher specificity and sensitivity.

At present, many studies have established a range of biomarkers for diagnosing and predicting the prognosis of HCC, including miRNAs, lncRNAs, and some specific genes. Shaker et al. (29) mentioned that lncRNA NEAT and miR-129-5p in plasma can be used as biomarkers for making diagnosis and estimating prognosis. According to Shimagaki et al. (30), plasma MFG-E8 can also be used as a biomarker for early diagnosis of HCC; it also has the ability to detect any recurrence in its early stage and to estimate the overall survival rate before liver resection. Chao et al. (31) proposed that the expression of lncRNA-D16366 was reduced, and it had an independent diagnostic value for HCC patients. Especially ST8SIA6-AS1 was also reported to be a great diagnostic indicator, and it may reach better diagnostic efficiency when combined with AFP in HCC patients (32). Our previous study found a high level of expression of lnc-MyD88 in early-stage HCC, and it participates in the occurrence and progression of HCC (23). In the study, we further explore the diagnostic value of lnc-MyD88 in the plasma of HCC patients, especially the differential expression of lnc-MyD88 in the three groups, namely, healthy subjects, LC patients, and HCC patients.

In our prior research, we found that lnc-MyD88 is significantly expressed in liver cancer tissues and can affect the growth and metastasis of HCC by affecting MyD88 (23). In the present study, lnc-MyD88 levels were significantly increased in the plasma of HCC patients in comparison with the plasma of LC patients and healthy subjects. Further ROC curve analysis confirmed that lnc-MyD88 has better sensitivity and specificity compared with AFP. Diagnostic performance analyses of lnc-MyD88 and AFP with their respective diagnostic values can help discriminate between HCC patients and healthy individuals; it indicates that the relative expression level of lnc-MyD88 can be used as a marker for HCC like AFP. In addition, diagnostic performance analyses of lnc-MyD88 and AFP demonstrating specific parameters can distinguish between HCC patients and LC patients. Therefore, lnc-MyD88 and AFP can also be used as effective tumor markers to distinguish HCC from LC, and the AUC value of lnc-MyD88 suggested that lnc-MyD88 had better diagnostic efficiency than AFP in the process of distinguishing HCC from LC. The result of multivariate analysis further identified that lnc-MyD88 had the best diagnostic efficiency among different laboratory investigations.

Our previous research verified that lnc-MyD88 had a positive correlation with MyD88, and it can influence the progression by mediating MyD88 expression. In the present study, the results showed that MyD88 expression was correlated with the infiltrations of eosinophils, T helper cells, Tcm, and pDCs. Moreover, the immune-related genes including chemokine genes, chemokine receptor genes, immunostimulatory genes, and immunoinhibitory genes all positively correlated with the MyD88 expression. Recent studies mentioned that eosinophils may contribute to the regulation of the tumor microenvironment (33, 34). Yan et al. (35) identified T helper 17 cell as an effective prognostic biomarker in HCC. The activation of pDCs could mediate the tolerance of T cells in human cancers (36). It indicated that lnc-MyD88 may have a correlation with immune infiltrations, and it may play an important role in the immunotherapy of HCC patients.

Our study also had many limitations. The major limitation of this study is that it neither investigated the prognosis of HCC patients nor explored whether lnc-MyD88 in plasma has a guiding role in the prognosis assessment of HCC. Secondly, the diagnostic value of lnc-MyD88 should be proven again by utilizing a new patient population. Especially, we need to enroll more patients with other types of virus infection to validate the diagnostic value of lnc-MyD88. These will be performed in our future work.

## Conclusion

The distinct expression of lnc-MyD88 is high in HCC and may be used as an innovative but promising diagnostic biomarker. It also had great potential to be an immunotherapeutic target for HCC patients.

## Data availability statement

The original contributions presented in the study are included in the article/supplementary material. Further inquiries can be directed to the corresponding authors.

## Ethics statement

The project was approved by the Ethics Committee of the hospital and written informed consent was obtained from each patient who enrolled in the study.

## Author contributions

ZW and PG contributions to the conception and work design; JJ and SX gathered the clinical samples and literature; ZW and AR created the diagrams and tables; PG and CZ completed the writing and editing of this manuscript. ZW, PG, WS, AR, JJ, SX, CX, CZ, and XQ completed the review and revision of this manuscript. All authors contributed to the article and approved the submitted version.
